# Two states of a light-sensitive membrane protein captured at room temperature using thin-film sample mounts

**DOI:** 10.1107/S2059798321011220

**Published:** 2022-01-01

**Authors:** Danny Axford, Peter J. Judge, Juan F. Bada Juarez, Tristan O. C. Kwan, James Birch, Javier Vinals, Anthony Watts, Isabel Moraes

**Affiliations:** a Diamond Light Source, Harwell Science and Innovation Campus, Didcot OX11 0DE, United Kingdom; b Biochemistry Department, University of Oxford, South Parks Road, Oxford OX1 3QU, United Kingdom; c National Physical Laboratory, Hampton Road, Teddington, London, United Kingdom; d Research Complex at Harwell, Rutherford Appleton Laboratory, Harwell Science and Innovation Campus, Didcot OX11 0FA, United Kingdom

**Keywords:** membrane proteins, microbial rhodopsin, archaerhodopsin, retinal, LCP, room temperature, synchrotron, proton transport, thin-film sample, photoreceptors, polymer films, lipidic cubic phase

## Abstract

Room-temperature diffraction methods are highly desirable for dynamic studies of biological macromolecules, since they allow high-resolution structural data to be collected as proteins undergo conformational changes. For crystals grown in lipidic cubic phase (LCP), an extruder is commonly used to pass a stream of microcrystals through the X-ray beam; however, the sample quantities required for this method may be difficult to produce for many membrane proteins. A more sample-efficient environment was created using two layers of low X-ray transmittance polymer films to mount crystals of the archaerhodopsin-3 (AR3) photoreceptor and room-temperature diffraction data were acquired. By using transparent and opaque polymer films, two structures, one corresponding to the desensitized, dark-adapted (DA) state and the other to the ground or light-adapted (LA) state, were solved to better than 1.9 Å resolution. All of the key structural features of AR3 were resolved, including the retinal chromophore, which is present as the 13-*cis* isomer in the DA state and as the all-*trans* isomer in the LA state. The film-sandwich sample environment enables diffraction data to be recorded at room temperature in both illuminated and dark conditions, which more closely approximate those *in vivo*. This simple approach is applicable to a wide range of membrane proteins crystallized in LCP and light-sensitive samples in general at synchrotron and laboratory X-ray sources.

## Abstract 2

High-resolution X-ray diffraction data were collected at room temperature for light- and dark-adapted states of the archaerhodopsin-3 photoreceptor using transparent and opaque polymer-film sample mounts.

## 1. Introduction

Room-temperature diffraction methods are highly desirable for dynamic studies of biological macromolecules, since they allow high-resolution structural data to be collected as proteins undergo conformational changes (Shoemaker & Ando, 2018 ▸; Ren *et al.*, 2020 ▸). Fixed-target approaches, in which crystals are loaded onto chips that can be scanned across the X-ray beam, have proven to be successful for soluble proteins (Martiel *et al.*, 2019 ▸; Mehrabi *et al.*, 2020 ▸). For membrane-protein crystals grown in lipidic cubic phase (LCP), an extruder is commonly used to pass a stream of microcrystals through the X-ray beam; however, this method requires a considerable amount of protein sample, the production of which may not be feasible for many membrane proteins (Weierstall *et al.*, 2014 ▸; Ren *et al.*, 2020 ▸). New approaches, including those using thin polymer films (Huang *et al.*, 2015 ▸; Axford *et al.*, 2016 ▸; Doak *et al.*, 2018 ▸; Lieske *et al.*, 2019 ▸), are therefore required to allow complete data sets to be obtained from small sample volumes, especially if the quantity of crystals is limited, without recourse to specialized sample-delivery mechanisms and data-collection environments.

The photoreceptor archaerhodopsin-3 (AR3) from the archaebacterium *Halorubrum sodomense* is a light-driven proton transporter. Like other microbial rhodopsins, it has seven transmembrane helices and a retinylidene chromophore created by the covalent conjugation of retinal to Lys226 via a Schiff base (Bada Juarez *et al.*, 2021 ▸). Absorption of a photon of appropriate wavelength initiates a highly ordered sequence of conformational changes (the photocycle) which results in the movement of one H^+^ ion from the cytoplasm to the extracellular medium (Chow *et al.*, 2010 ▸; Saint Clair *et al.*, 2012 ▸). Interest in the transporter has grown over the past decade, primarily because of its widespread applications in optogenetics (Guru *et al.*, 2015 ▸; McIsaac *et al.*, 2015 ▸); mutants of AR3 are now routinely used as silencers of mammalian neurons (El-Gaby *et al.*, 2016 ▸; Rost *et al.*, 2017 ▸) and as fluorescent sensors of transmembrane potential (Kralj *et al.*, 2012 ▸; McIsaac *et al.*, 2014 ▸).

AR3, along with many other microbial rhodopsins, has a desensitized, dark-adapted (DA) state which is formed from the resting, light-adapted (LA) state in the absence of light. The DA state is characterized by a thermal equilibrium between at least two conformations: one possessing all-*trans* retinal, in common with the LA state, and one with 13-*cis* 15-*syn* retinal (Saint Clair *et al.*, 2012 ▸). Our understanding of the transition between the DA and LA states has recently been advanced with new atomic resolution cryo crystal structures (Bada Juarez *et al.*, 2021 ▸).

In this study, microcrystals of AR3 were prepared for data collection in light and dark environments by sandwiching them between 13 µm thick sheets of polymer film with high X-ray transmittance (Axford *et al.*, 2016 ▸). Transparent cyclic olefin polymer (COP) and opaque black Kapton provided light-permissive and light-tight containment, respectively (Supplementary Fig. S1), and enabled diffraction data to be collected at room temperature, significantly reducing the risk of crystal dehydration. The opaque sample mount enabled AR3 desensitization to the DA state, which was maintained and captured at 1.9 Å resolution in the diffraction data. This research not only demonstrates that AR3 can convert from LA to DA in a crystalline environment, but also that conversion to the DA state can take place at room temperature, with no apparent input of energy. Here, we show that the thin-film mounts are suitable for collecting high-resolution room-temperature diffraction data for membrane proteins, for which the supply of crystals is limited. This approach also provides a simple and accessible method for the X-ray scattering analysis of light-sensitive samples.

## 2. Materials and methods

### 2.1. Protein expression and purification

*H. sodomense* (ATCC 33755) cells were purchased from LGC Standards Ltd (Teddington, UK) and were grown without any genetic modification. Initially, cells were grown in 4 × 25 ml liquid culture medium at pH 7.4 with high salt concentration [125 g NaCl, 160 g MgCl_2_, 0.13 g CaCl_2_, 5 g K_2_SO_4_, 1 g bacteriological peptone (Oxoid, UK), 1 g yeast extract (Melford, UK) and 2 g soluble starch (Sigma) per litre] at 45°C with shaking at 170 rev min^−1^ for 5–7 days or until the OD_600_ reached 1.2. Fresh culture medium (of identical composition) was inoculated from the 25 ml cultures and was incubated at 45°C with shaking at 170 rev min^−1^ for approximately three weeks until an OD_600_ of 1.9 was reached.

Cells were harvested by centrifugation (8000*g*, 30 min, 4°C) and the pellets were resuspended in 4 *M* NaCl. DNAse I (Sigma, UK) was added and the solution was stirred for 2 h before being manually homogenized using a Potter–Elvehjem homogenizer. The preparation was dialyzed overnight in 0.1 *M* NaCl and centrifuged (70 000*g*, 50 min, 4°C). Sucrose-density gradient ultracentrifugation (110 000*g* for 15 h at 15°C) was used to isolate the AR3-rich membrane, using a step gradient consisting of layers of 4 ml sucrose solution at densities of 30, 40, 50 and 60%(*w*/*v*). The lower band with a pink/purple colour was collected, and the sucrose remaining in the sample was removed through overnight dialysis against distilled water. The sample was then further centrifuged (70 000*g*, 50 min, 4°C) and the pellet was resuspended in distilled water to a final concentration of 20 mg ml^−1^ and stored at 4°C prior to crystallization. A yield of 7 mg protein was produced per litre of cell culture and, using SDS-polyacrylamide gel electrophoresis, the AR3 content was estimated to be 78 ± 2%(*w*/*w*) of the total protein.

### 2.2. Crystallization procedure

The crystallization process for AR3 has been reported previously (Bada Juarez *et al.*, 2021 ▸). Briefly, LCP was prepared by mixing the non-delipidated protein sample with monoolein (Nu-Check Prep Inc., Elysian, Minnesota, USA) in a 2:3(*v*:*v*) ratio using two gas-tight Hamilton syringes connected by an SPT Labtech syringe coupler (Caffrey & Cherezov, 2009 ▸). All crystallization procedures were performed under dim light and the growing crystals were incubated at 20°C in the dark. Crystals grew from a precipitant solution consisting of 33%(*v*/*v*) polyethylene glycol 600 (Fluka Analytical), 100 m*M* MES buffer pH 5.5, 150 m*M* NaCl, 150 m*M* Ca^2+^ to form rods with typical sizes of 15–20 µm in the shortest dimensions and 100–150 µm in length.

### 2.3. Data collection

**Figure 1:**
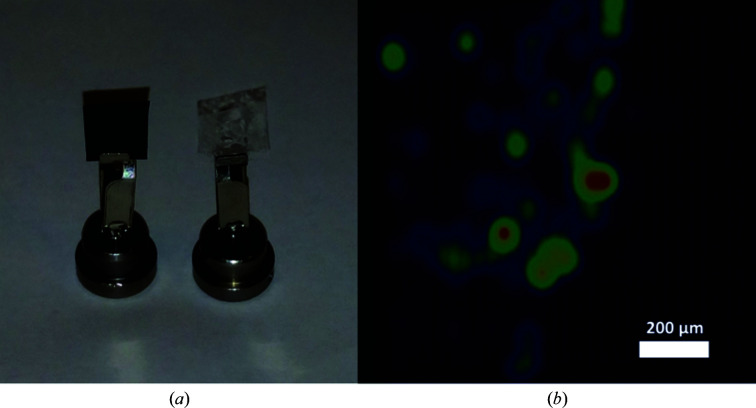
Film sample mounts and diffraction screening. (*a*) Polymer film sample mounts held on DiffraX pins ready for data collection: black Kapton film (left) and transparent COP (right). (*b*) Results of a diffraction raster scan on the dark Kapton sample preparation as a heat map indicating the locations of crystals. The colour map (blue through yellow to red) indicates an increasing number of Bragg spots as determined by the *DIALS* software package (Gerstel *et al.*, 2019 ▸).

For the LA structure, a small amount of crystal-containing lipidic sponge phase was sandwiched between two 13 µm thick transparent cyclic olefin polymer (COP) films (Zeon, Dusseldorf, Germany) separated by a 50 µm thick adhesive spacer containing a 5 mm diameter aperture (4titude, Wotton, UK; Fig. 1 ▸ *a*; Supplementary Figs. S1 and S2). This ‘sandwich’ of crystals was cut to a ∼7 × 7 mm square using a scalpel and was mounted by hand onto the beamline goniometer using a DiffraX pin (Molecular Dimensions, Newmarket, UK) as described previously (Axford *et al.*, 2016 ▸). For the DA structure, the COP film was replaced by light-impermeable 12 µm black Kapton (Dupont; Fig. 1 ▸ *a*), which has a maximum transmittance of <0.6% for all wavelengths in the range 200–640 nm and a maximum transmittance of <1.5% for all wavelengths in the range 640–840 nm (Dupont, personal communication). These samples were stored at room temperature in the dark for at least 30 min before data collection to allow the protein in the opaque Kapton film sandwich to change conformation to the DA state.

The final LA data set was formed by merging data from three crystals. The first was oriented parallel to the spindle axis and a helical collection of 50° rotation was performed while translating along the length of the crystal, with the X-ray beam focused to 8 × 8 µm; the second and third crystals were orientated orthogonally to the spindle axis and a defocused beam, ∼40 × 8 µm, was used to approximate the sample cross-section during rotation. The nature of the thin-film sandwich forces the rod-shaped crystals of AR3 to align parallel with the plane of the film. In order to collect complete data, the second and third crystals were rotated up to 60° and 50° away from face-on and with total angular ranges of 30° and 50°, respectively. The latter crystal received the largest X-ray dose, which was calculated to be 80 kGy using *RADDOSE*3*D* (Bury *et al.*, 2018 ▸).

To collect the data for the DA structure, crystals were mounted between two layers of black Kapton. A raster scan was performed with an attenuated beam (one third of the per-image dose used for data collection) to locate the crystals beneath the opaque film (Fig. 1 ▸ *b*). Small wedges of data were then collected from each crystal ‘hit’ and the starting angle was varied to maximize sampling of reciprocal space. 18 partial data sweeps (10–15°) were collected in total using a defocused 30 × 30 µm beam, with the maximum dose per wedge calculated to be 15 kGy.

### 2.4. Data processing and structure solution

All diffraction data were integrated with *DIALS* (Winter *et al.*, 2018 ▸). For the three LA partial data sets *AIMLESS* was used to merge and scale the measurements (Evans *et al.*, 2011 ▸). For the DA data, *xia*2.*multiplex* was used to merge and scale the 18 partial data sets together. A summary of the data-processing statistics is presented in Table 1 ▸. Phases for the AR3 cryo structures were obtained by molecular replacement with *Phaser* (version 2.7.17; McCoy *et al.*, 2007 ▸) from the *CCP*4 suite (version 7.0.066; Winn *et al.*, 2011 ▸) using the 3.4 Å resolution structure of archaerhodopsin-1 (PDB entry 1auz; Enami *et al.*, 2002 ▸). The AR3 structures were refined using *Phenix* (Liebschner *et al.*, 2019 ▸) and structural models were built using *Coot* (Emsley *et al.*, 2010 ▸).

## 3. Results and discussion

Two AR3 structures were solved, the first to 1.85 Å resolution (PDB entry 6s63) in the opaque-film sandwich and the second to 1.9 Å resolution (PDB entry 6guz) in the transparent-film sandwich. Data-processing statistics are reported in Table 1 ▸. Although the tolerance of the crystals to X-ray dose is lower at room temperature than under cryo conditions, the structures are sufficiently well resolved to allow all of the key structural elements of AR3 to be identified. Data completeness is somewhat compromised at around ∼90% due to the enforced systematic orientation of the rod-shaped crystals when squeezed between the two film sheets, creating what is effectively a two-dimensional sample mount (Supplementary Fig. S2). Although it might be possible to limit the amount of flattening of the sample to maintain a greater range of crystal orientations, this would have led to a much longer beam path through the LCP, resulting in higher background scatter, thereby compromising the diffraction resolution.

**Figure 2:**
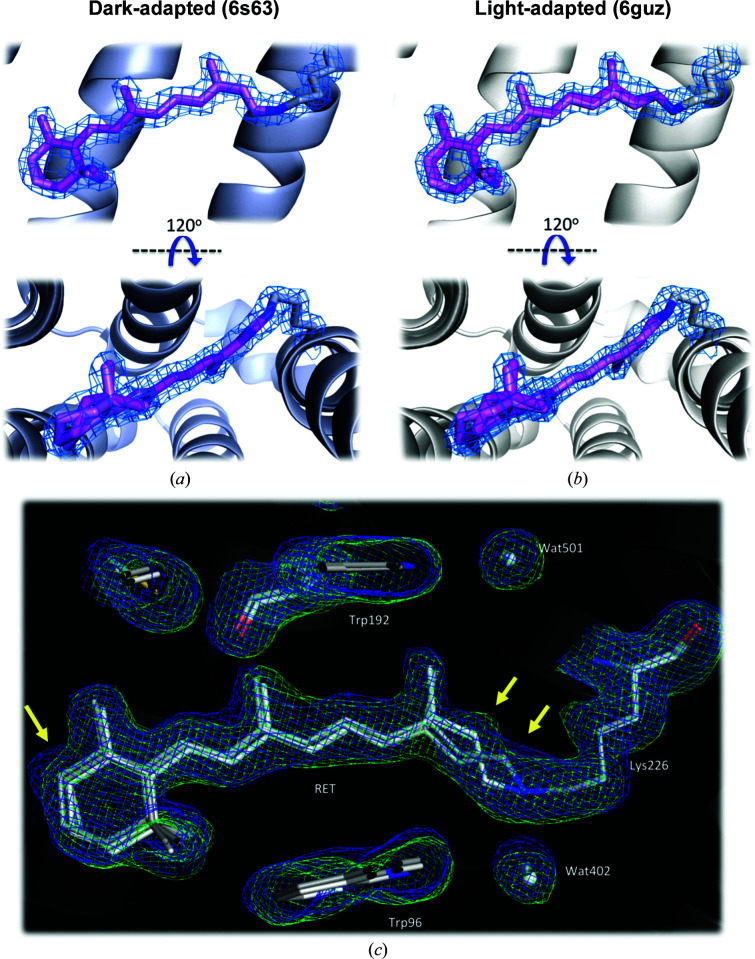
Retinal-binding pocket. Conformations of retinal (coloured in light and dark pink) in (*a*) the DA state (PDB entry 6s63) and (*b*) the LA state (PDB entry 6guz) of AR3. The blue mesh in (*a*) and (*b*) around retinal and Lys226 represents the 2*F*_obs_ − *F*_calc_ electron-density map contoured at 1.5σ. Omit maps are shown in Supplementary Fig. S3. (*c*) Overlay of composite omit maps (2*F*_obs_ − *F*_calc_) of the DA (PDB entry 6s63; grey) and LA (PDB entry 6guz; white) states for retinal and selected amino acids from the surrounding binding pocket. The electron-density maps (LA, blue mesh; DA, green mesh) for the two structures were created using *Phenix* and are contoured at 1.0σ. The differences in the distributions of electron density surrounding the β-ionone ring and the C_13_=C_14_ bond are indicated by yellow arrows. Water molecules are represented by grey and white spheres. All images were created using *PyMOL*.

All of the major structural features visible in the high-resolution cryo crystal structures were also resolved at room temperature, including the retinylidene chromophore, which is formed from the covalent conjugation of retinal to Lys226, and the conversion of the N-terminal Gln7 to a pyroglutamyl group (Bada Juarez *et al.*, 2021 ▸; Hoi *et al.*, 2021 ▸). The most striking difference between the two room-temperature structures is the contrasting retinal conformation, which is resolved as the 13-*cis* isomer in PDB entry 6s63 but as the all-*trans* isomer in PDB entry 6guz, consistent with these structures representing the DA and LA states, respectively (Fig. 2 ▸; Supplementary Fig. S3). Two different retinal conformers are modelled for each structure and the greatest apparent movement is around the β-ionone ring. Although the room-temperature structures are at lower resolution, the conformations of retinal observed here are broadly consistent with those in the previously reported cryo structures (Bada Juarez *et al.*, 2021 ▸). The 1.3 Å resolution DA structure (PDB entry 6gux) has both 13-*cis* and all-*trans* retinal modelled with relative occupancies of 70:30, and the lower resolution of the corresponding room-temperature structure (PDB entry 6s63) is likely to explain why only the 13-*cis* isomer could be modelled. Two forms of the all-*trans* isomer were modelled into the 1.07 Å resolution LA cryo structure (PDB entry 6s6c), consistent with the room-temperature LA structure reported here (PDB entry 6guz; Bada Juarez *et al.*, 2021 ▸).

**Figure 3:**
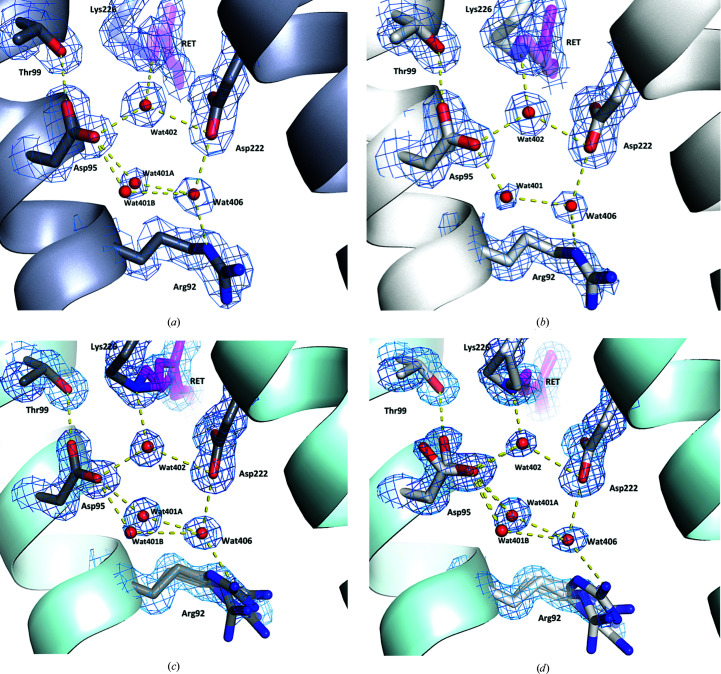
Structures of the pentagonal hydrogen-bond networks in AR3 at room and cryogenic temperatures. The pentagonal hydrogen-bond networks in the DA (PDB entry 6s63) and LA (PDB entry 6guz) states of AR3 at room temperature are shown in (*a*) and (*b*), respectively, while in (*c*) and (*d*) the pentagonal hydrogen-bond networks in the DA (PDB entry 6gux) and LA (PDB entry 6s6c) states of AR3, respectively, at cryogenic temperatures are shown. Water molecules are shown as red spheres and predicted hydrogen bonds are shown as dashed yellow lines. The blue mesh represents the 2*F*_obs_ − *F*_calc_ electron-density map contoured at 1.5σ. Omit maps are shown in Supplementary Fig. S4.

Analysis of the protein structure surrounding the Schiff base identifies further differences between the two room-temperature structures and their cryo counterparts. All of the side chains in this region are resolved in only one conformation in the two AR3 structures reported here (Fig. 3 ▸); however, in the LA cryo structure (PDB entry 6s6c) the Asp95 side chain is resolved as two rotamers, consistent with partial breakage of the hydrogen bond to Thr99. Arg92, which is resolved as four rotamers in the two cryo structures, is only resolved in one position in the two room-temperature structures (Bada Juarez *et al.*, 2021 ▸).

There are several bound water molecules within the half channels that link the Schiff base to the intracellular and extracellular sides of the membrane. In general, the positions of these molecules do not vary significantly between the DA and LA states reported here. One exception is Wat401, which is hydrogen-bonded to Asp95 and to Wat406 (Fig. 3 ▸) and which is modelled in two positions in the DA structure (PDB entry 6s63) but in only one position in the LA structure (PDB entry 6guz). The single Wat401 position in PDB entry 6guz is at the approximate midpoint between the two positions in PDB entry 6s63, and the relatively high *B* factor (23.64 Å^2^) suggests that this molecule is highly disordered.

Supplementary Table S1 shows the *B* factors for Wat401 and the neighbouring atoms in the LA and DA structures. There is no significant difference in the apparent order of the adjacent Wat406; however, the *B* factor of the OD2 atom of Asp95, to which Wat401 is hydrogen bonded, is higher in the DA state than in the LA state. In contrast, there is little difference in the *B* factors of the Asp95 OD1 atom, which may interact directly with the Thr99 side chain and indirectly with the Schiff base N atom via Wat402. The disorder observed in Wat401 is consistent with previous Fourier-transform infrared spectroscopy experiments, which have suggested greater movement of Wat401 in AR3 than in bacteriorhodopsin (Saint Clair *et al.*, 2012 ▸). Wat401 is implicated in determining the p*K*_a_ of the equivalent Asp side chain in bacteriorhodopsin (Chang *et al.*, 1988 ▸), and it is possible that a weaker hydrogen bond between Wat401 and Asp95 in the LA form reflects a higher p*K*_a_ for the side chain than in the DA form (Bada Juarez *et al.*, 2021 ▸).

## 4. Conclusion

Together these two structures show that high-resolution diffraction data may be obtained at room temperature using polymer films, and that the two different light- and dark-adapted states may be differentiated, despite the increased *kT* relative to cryogenic conditions. This simple method provides an alternative to freeze-quenching approaches that rely on trapping the conformation of the protein by shifting it far from its working temperature. The use of a light-tight sample support meant that the beamline could be operated in a completely normal configuration, rather than under ‘dark-room’ conditions that would require the blocking of all ambient light. Furthermore, the practicality of using pin-based sample mounts allows the method to be used on regular goniometers, including those used on laboratory X-ray sources.

## Supplementary Material

PDB reference: light-adapted archaerhodopsin-3, 6guz

PDB reference: dark-adapted archaerhodopsin-3, 6s63

Supplementary Figures and Table. DOI: 10.1107/S2059798321011220/nz5004sup1.pdf

## Figures and Tables

**Table 1:** Data-collection and refinement statistics Values in parentheses are for the highest resolution shell.

PDB code	6s63	6guz
Data collection		
Temperature (K)	293	293
No. of crystals	18	3
Space group	*P*2_1_2_1_2_1_	*P*2_1_2_1_2_1_
*a*, *b*, *c* (Å)	45.91, 48.35, 105.36	46.25, 48.30, 104.84
α, β, γ (°)	90, 90, 90	90, 90, 90
Wavelength (Å)	0.96862	0.96862
Resolution range (Å)	105.36–1.85 (1.88–1.85)	52.42–1.90 (1.99–1.90)
No. of unique observations	18417 (876)	20412 (1214)
Completeness (%)	89.2 (88.1)†	91.1 (93.7)
Multiplicity	8.5 (5.6)	5.0 (4.9)
*R*_p.i.m._ ‡	0.126 (0.623)	0.084 (0.338)
*R*_meas_ §	0.401 (1.380)	0.199 (0.802)
CC_1/2_	0.995 (0.316)	0.984 (0.675)
Mean *I*/σ(*I*)	6.6 (1.3)	4.9 (2.1)
Wilson *B* factor (Å^2^)	21.6	17.4
Refinement		
Resolution range (Å)	52.68–1.85	35.52–1.90
No. of observations (total/test set)	17485/970	16103/818
Completeness (%)	89.2	88.2
*R*_work_/*R*_free_ (%)	0.18/0.19	0.18/0.22
No. of atoms		
Protein	3811	3788
Ligand/ion	435	370
Waters	73	56
Average *B*, all atoms (Å^2^)	26.54	21.78
R.m.s. deviations		
Bond lengths (Å)	0.004	0.008
Bond angles (°)	0.951	1.462
Ramachandran plot		
Outliers (%)	0.0	0.0
Allowed (%)	1.79	1.79
Favoured (%)	98.21	98.21

† See Section 3 for an explanation of the relatively low completeness.

‡ *R*_p.i.m._ is the multiplicity-weighted, precision-indicating merging *R* factor for comparing symmetry-related reflections (Weiss & Hilgenfeld, 1997 ▸).

§ *R*_meas_ is the redundancy-independent multiplicity-weighted *R* factor for comparing symmetry-related reflections (Diederichs & Karplus, 1997 ▸).
