# Supplementary material for: Two states of a light-sensitive membrane protein captured at room temperature using thin-film sample mounts
Source: Acta Crystallogr D Struct Biol. 2022 Jan 1;78(Pt 1):52–8. doi: 10.1107/S2059798321011220 (PMC8725165; doi:10.1107/S2059798321011220)
Supplement: Supplementary file 1 [file d-78-00052-sup1.pdf]

Acta Cryst D STRUCTURAL BIOLOGY

**Volume 77 (2021)**

**Supporting information for article:**

# Two states of a light-sensitive membrane protein captured at room temperature using thin-film sample mounts

**Danny Axford, Peter J. Judge, Juan F. Bada Juarez, Tristan O. C. Kwan, James Birch, Javier Vinals, Anthony Watts and Isabel Moraes**

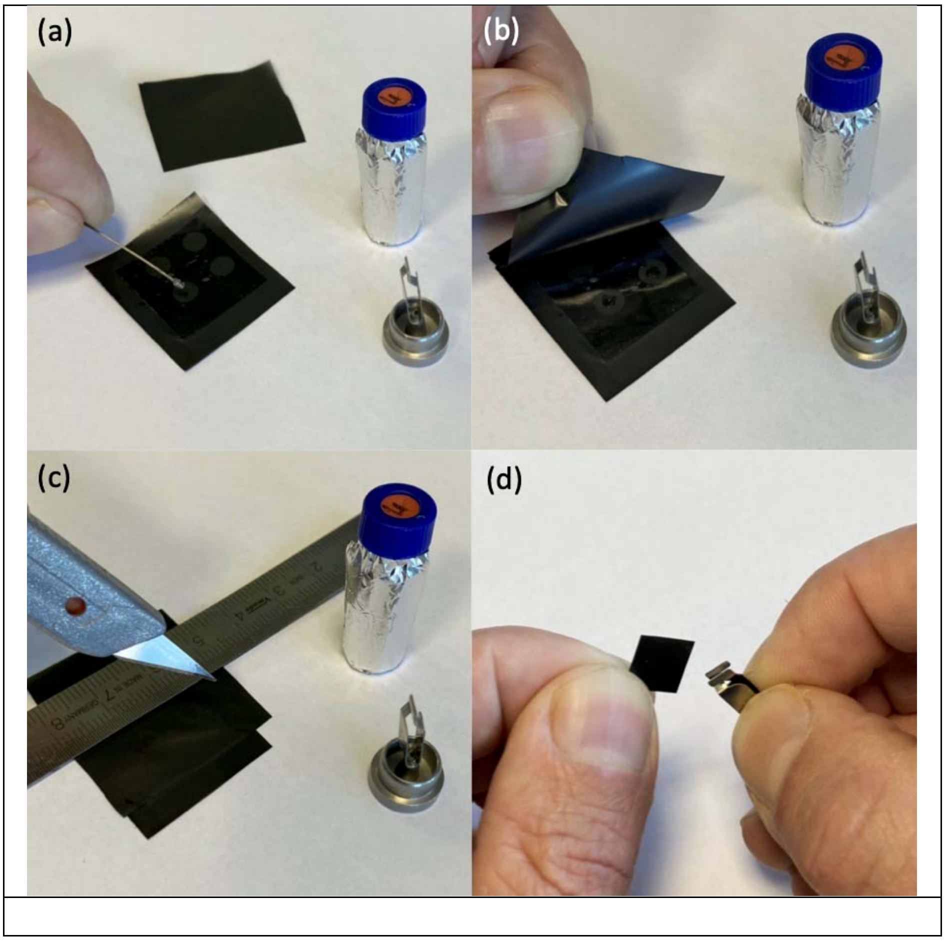


**Figure S1** Preparation of film sandwich using Kapton® 50B film. Materials include two sections of film, one with adhesive spacer added and backing layer removed, vial containing sample and a tweezer pin. The crystal-containing LCP is transferred from vial to spacer aperture on lower film using a needle (a). The top layer of film is added and pressed down (b). Individual apertures are cut out to size using a scalpel (c). A single-aperture section of the film sandwich is clasped by the tweezer pin ready for data collection (d).

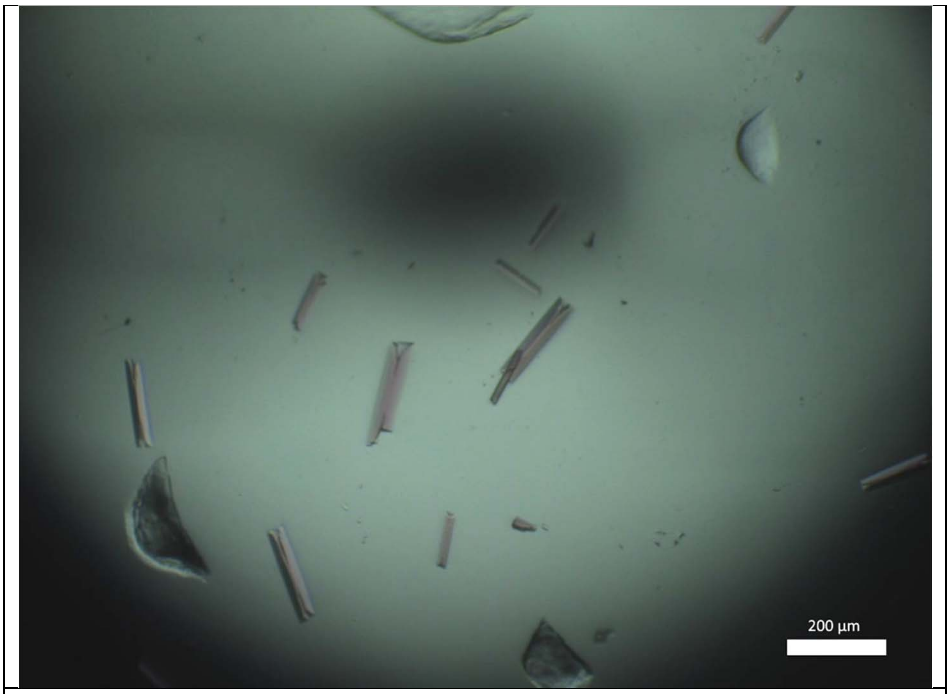


**Figure S2** On-beamline image of AR3 crystals sandwiched between two sheets of Cyclic Olefin Polymer film shortly before data collection. Viewing system consisted of Optem 10x M Plan APO infinity corrected objective (Excelitas, Waltham MA, US), Qioptiq Fetura+ zoom lens (Excelitas, Waltham MA, US) at x1 magnification and a Manta G-125C CCD camera (Allied Vision, Stadtroda, Germany).

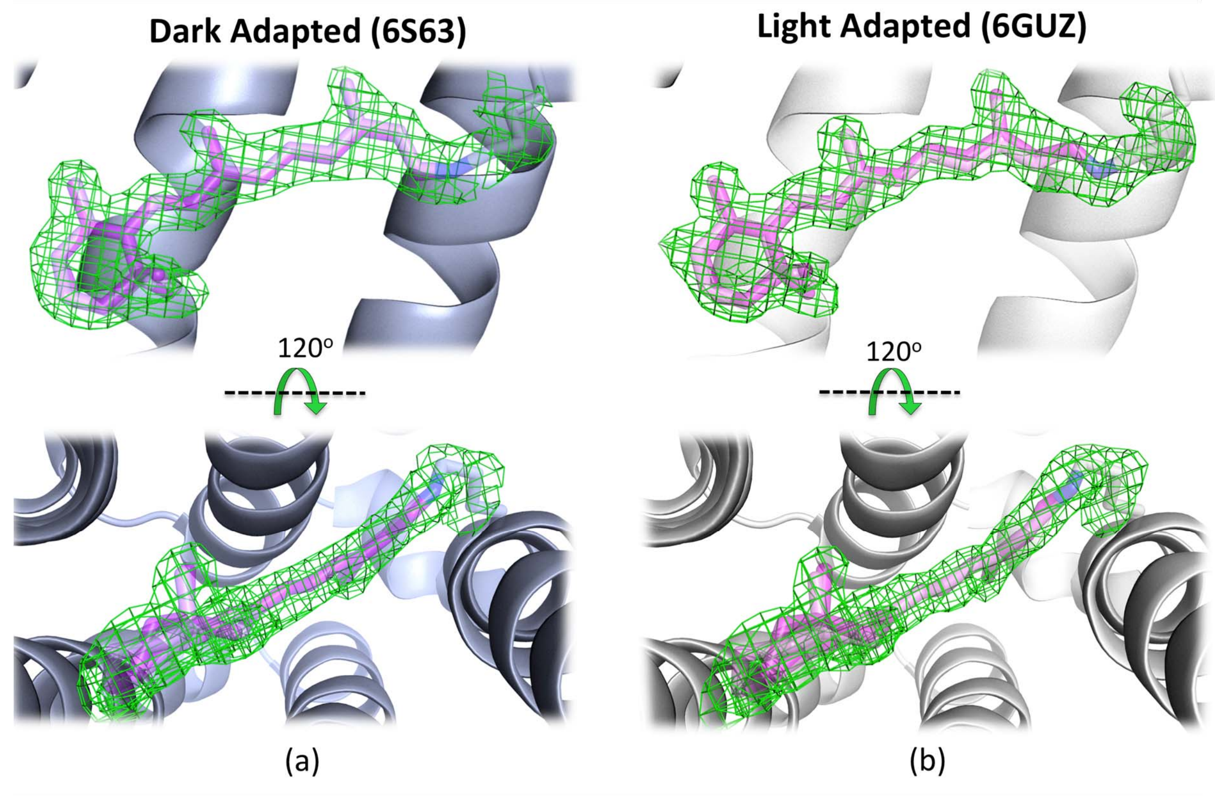


**Figure S3** Validation of the different conformations of retinal and Lys226 Omit electron density maps ($F_{obs}$-$F_{calc}$) of (a) the DA (6S63) and (b) LA (6GUZ) states of AR3. Here, the retinal and Lys226 were removed during the diff map production. The omit $F_{obs}$-$F_{calc}$ maps are represented by the green mesh (positive density) and were contoured at ±3σ.

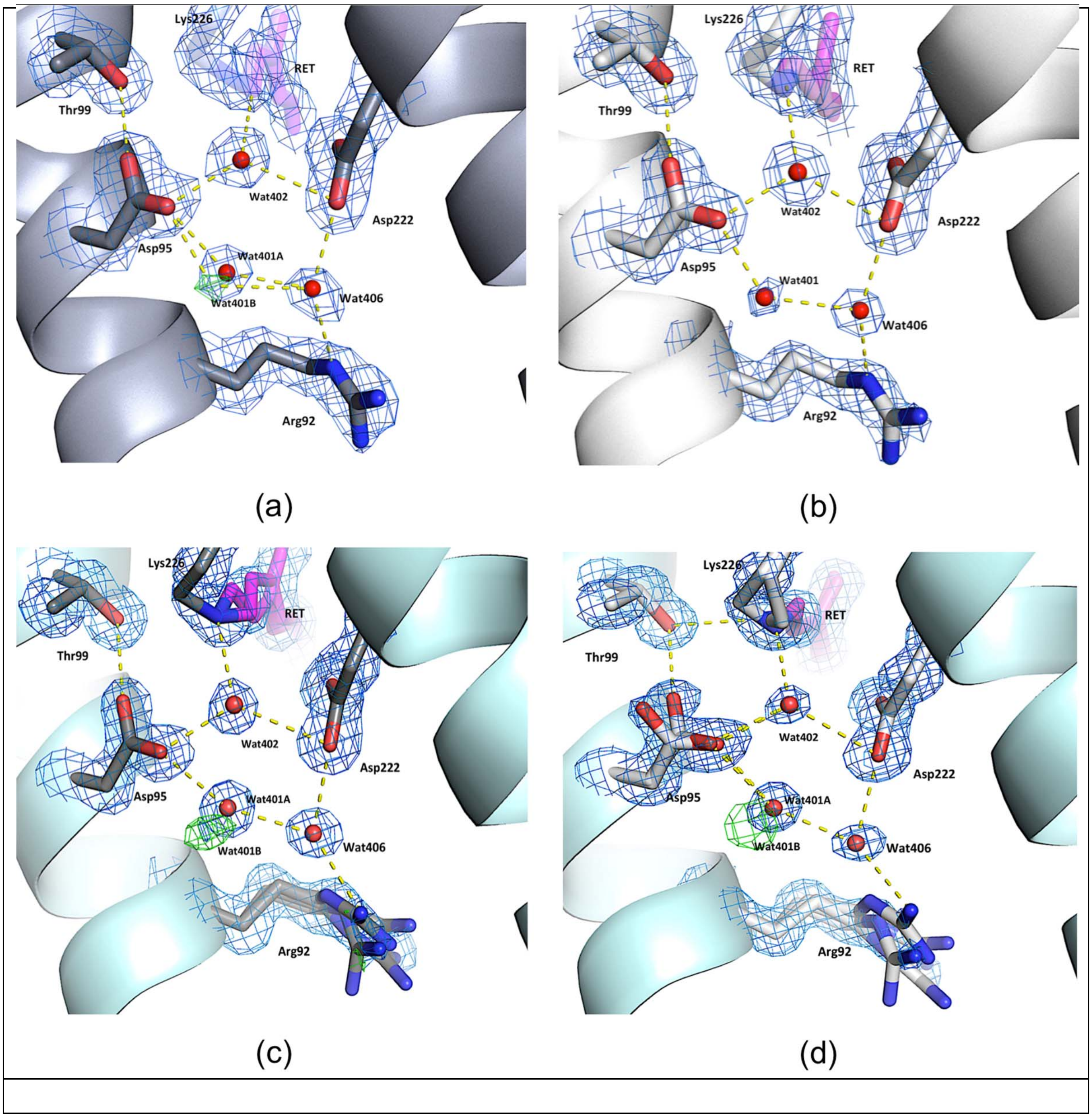


**Figure S4** Validation of the Wat401 positions by omit electron density maps ($mF_{obs}$-$F_{calc}$). The blue mesh represents the $2F_{obs}$-$F_{calc}$ electron density maps contoured at 1.5σ and the green mesh represents the positive density contoured at 3.5σ. Panels (a) and (b) show the pentagonal H-bond network for the DA (6S63) and LA (6GUZ) states of AR3 at room temperature. Panels (c) and (d) show the pentagonal H-bond network for the DA (6GUX) and LA (6S6C) states of AR3 at cryogenic temperatures. Water molecules are shown as red spheres and predicted H-bonds are shown as dashed yellow lines.

**Table S1** B factors (measured in Å$^2$) for Wat401 and the neighbouring atoms in the LA and DA structures. Note that Wat401 is modelled in two positions in the DA structure and the occupancy of each position is given in parentheses. Atom OD2 of Asp95 is understood to be H-bonded to Wat401. Atom OD1 of Asp95 may be able to form H-bonds to Thr99 and to Wat402.

| | **B factors of selected atoms (Å$^2$)** | | | |
|---|---|---|---|---|
| | Asp95 OD1 | Asp95 OD2 | Wat401(A/B) | Wat406 |
| **6GUZ (LA)** | 15.21 | 11.33 | 23.64 | 22.62 |
| **6S63 (DA)** | 14.15 | 15.10 | 16.82 (70%) / 15.21 (30%) | 22.60 |
